# Increased phytotoxic O_3_ dose accelerates autumn senescence in an O_3_-sensitive beech forest even under the present-level O_3_

**DOI:** 10.1038/srep32549

**Published:** 2016-09-07

**Authors:** Mitsutoshi Kitao, Yukio Yasuda, Yuji Kominami, Katsumi Yamanoi, Masabumi Komatsu, Takafumi Miyama, Yasuko Mizoguchi, Satoshi Kitaoka, Kenichi Yazaki, Hiroyuki Tobita, Kenichi Yoshimura, Takayoshi Koike, Takeshi Izuta

**Affiliations:** 1 Department of Plant Ecology, Forestry and Forest Products Research Institute, Matsunosato 1, Tsukuba 305-8687, Japan; 2 Tohoku Research Center, Forestry and Forest Products Research Institute, Nabeyashiki 92-25, Morioka 020-0123, Japan; 3 Kansai Research Center, Forestry and Forest Products Research Institute, Nagaikyutaroh 68, Kyoto 612-0855, Japan; 4 Hokkaido Research Center, Forestry and Forest Products Research Institute, Hitsujigaoka 7, Sapporo 062-8516, Japan; 5 Department of Forest Science, Hokkaido University, Sapporo 060-8589, Japan; 6 Institute of Agriculture, Tokyo University of Agriculture and Technology, Fuchu, Tokyo 183-8509, Japan

## Abstract

Ground-level ozone (O_3_) concentrations are expected to increase over the 21^st^ century, especially in East Asia. However, the impact of O_3_ has not been directly assessed at the forest level in this region. We performed O_3_ flux-based risk assessments of carbon sequestration capacity in an old cool temperate deciduous forest, consisting of O_3_-sensitive Japanese beech (*Fagus crenata*), and in a warm temperate deciduous and evergreen forest dominated by O_3_-tolerant Konara oak (*Quercus serrata*) based on long-term CO_2_ flux observations. On the basis of a practical approach for a continuous estimation of canopy-level stomatal conductance (G_s_), higher phytotoxic ozone dose above a threshold of 0 uptake (POD0) with higher G_s_ was observed in the beech forest than that in the oak forest. Light-saturated gross primary production, as a measure of carbon sequestration capacity of forest ecosystem, declined earlier in the late growth season with increasing POD0, suggesting an earlier autumn senescence, especially in the O_3_-sensitive beech forest, but not in the O_3_-tolerant oak forest.

Ground-level ozone (O_3_) levels are increasing globally, and further increases are expected over the 21^st^ century, especially in East Asia^1^,^2^,^3^,^4^. O_3_ is a detrimental air pollutant for vegetation, which reduces the photosynthetic rate, increases the respiration rate, and accelerates leaf senescence^5^. Accordingly, global limitation in the carbon (C) sink strength of forests by future-level O_3_ has been simulated through modeling using photosynthetic responses to O_3_ in young trees^6^. However, the impact of O_3_ on the C sequestration capacity has not been directly assessed in the temperate forests in East Asia on the basis of long-term observations.

In this sense, a unique opportunity to assess the C sequestration capacity of mature forests in East Asia would be on the basis of long-term CO_2_ flux observations^7^, as it was previously done with flux-based O_3_ assessment for Mediterranean forests^8^. Such observations could be done in Japan, which is located on the edge of East Asia, where a continuous increase in O_3_ concentration has been observed due to pollutants advected mainly from East Asia^4^,^9^,^10^,^11^. Since 2000, the flux tower sites of Forestry and Forest Products Research Institute (FFPRI) have been monitoring CO_2_, energy, and water vapor fluxes in several forests, including cool and warm temperate deciduous forests, with different tree species across Japan. Although Mediterranean forests mitigate O_3_ uptake via stomatal closure due to dry conditions during summer^12^, humid climate in Japanese temperate forests may promote stomata opening even in summertime, resulting in higher risk of O_3_ dose. Therefore, O_3_ flux-based risk assessments with Japanese forests would be of interest.

O_3_ flux-based risk assessment is more essential for evaluating the physiological effects of O_3_ on plants than the O_3_ exposure-based risk assessment^13^,^14^. To estimate O_3_ fluxes at the forest level, canopy-level stomatal conductance is required, as well as O_3_ concentrations over the forest, which is generally estimated by the Penman–Monteith (P–M) equation based on energy and water flux over a forest^13^. However, this approach is valid only when the entire evaporation process occurs through stomatal transpiration. Contrary to evergreen Mediterranean forests^8^,^15^, it is difficult to conduct a continuous estimation of the canopy-level stomatal conductance using the P–M approach in both cool and warm temperate deciduous forests, which have regular rainfall and a period when the canopy is not closed in spring and autumn^16^. We have developed a practical approach, which combines the P–M approach with a photosynthesis-dependent stomatal model for the continuous estimation of stomatal conductance over the canopy of the temperate deciduous forests^17^.

On the basis of the practical approach, we performed long-term O_3_ flux-based risk assessments on photosynthetic CO_2_ uptake in a cool temperate deciduous forest, consisting purely of deciduous broadleaf tree species, Japanese beech (*Fagus crenata* Blume)^18^, and a warm temperate mixed deciduous and evergreen broadleaf forest, dominated by deciduous broadleaf tree species, Konara oak (*Quercus serrata* Thunb. ex. Murray) (Fig. 1)^19^. Japanese beech is O_3_ sensitive, but Konara oak is relatively O_3_ tolerant among the Japanese tree species based on the growth responses under O_3_ exposure^20^,^21^. We found that light-saturated gross primary production (GPP), as a measure of C sequestration capacity of forest ecosystem, declined earlier with increasing phytotoxic ozone dose above a threshold of 0 uptake (POD0), especially in the late growth season in the O_3_-sensitive beech forest even under the present level O_3_, but not in the O_3_-tolerant oak forest.

## Results

### O_3_ exposure and O_3_ dose in the cool temperate beech forest and the warm temperate oak forest

**Figure 2:**
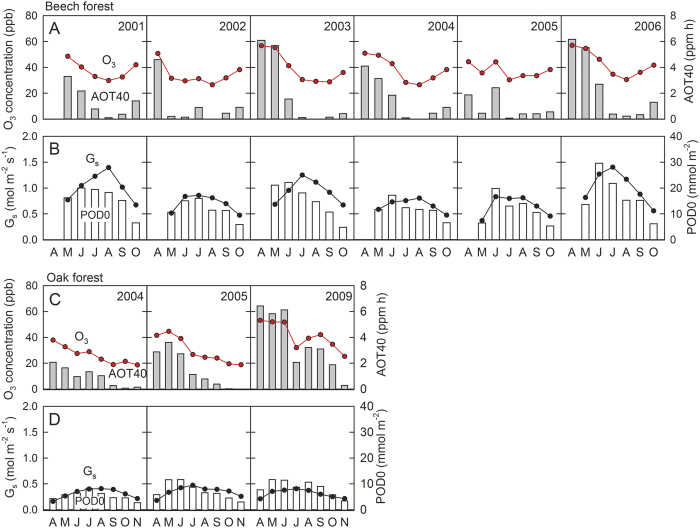
The mean of daytime O_3_ concentration above the canopy (red circles), AOT40 (gray bars) (**A**), mean of canopy stomatal conductance (G_s_, black circles) and phytotoxic O_3_ dose above the threshold of 0 (POD0, white bars) (**B**) of the beech forest for each month during the growth period from 2001 to 2006 and of the oak forest (**C**,**D**, respectively) during the growth periods of 2004, 2005, and 2009. O_3_ concentration above the canopy was estimated by the multiple regression model for each forest site using O_3_ and NO_2_ concentrations at the adjacent air pollution station, as well as air temperatures both at the station and the forest site. G_s_ was estimated from the modified Ball–Woodrow–Berry model. The datasets from 2006 to 2008 are unavailable at present. Daytime was defined as the photoperiod when the photon flux density is >0.

O_3_ exposure and O_3_ dose showed substantial seasonal variations at both forest sites. As the cool temperate forest almost purely consists of deciduous beech trees, canopy-level stomatal conductance (G_s_) and POD0, which are defined as integrated O_3_ fluxes, were detectable only from May to October during the growth period^18^. In contrast, as the warm temperate forest was a mixed deciduous and evergreen forest, G_s_ was detectable throughout the year^17^,^19^. We detected G_s_ from April to November corresponding to the growth period of the dominant deciduous tree species, *Q. serrata* (Konara oak)^17^. O_3_ exposure, demonstrated by daytime O_3_ concentration and AOT40 (O_3_ exposure index: accumulated ozone exposure over a threshold of 40 ppb), was relatively higher in spring from April to June in both sites (Fig. 2A,C), whereas G_s_ increased from spring to summer and then decreased toward autumn along with leaf senescence (Fig. 2B,D). Mean values of O_3_ concentration during the growth season (May to October for the beech forest and April to November for the oak forest) were averaged across years (Fig. 2): 38.5 ± 2.9 ppb (mean ± SD) at Appi forest site and 32.9 ± 7.9 ppb at Yamashiro forest site, respectively. There was no significant difference in mean O_3_ concentration between the two forests (p = 0.15, using t-test). The monthly POD0 reached its maximum generally in June throughout the 6 years (2001–2006) in the beech forest (Fig. 2B). As for the oak forest, O_3_ exposure was relatively higher in spring, but G_s_ peaked in July in all the 3 years, leading to a variation in the timing, reaching maximum POD0 (Fig. 2D). The integrated AOT40 values for the growth season in the beech forest (May–October) were 8.2, 2.7, 8.0, 6.5, 4.4, and 10.6 ppm h in 2001, 2002, 2003, 2004, 2005, and 2006, respectively (Fig. 2A). In contrast, a relatively higher integrated AOT40 values for the growth season (April–November) were observed in the oak forest: 8.0, 11.9, and 29.3 ppm h in 2004, 2005, and 2009, respectively (Fig. 2C).

### Relationship between the light-saturated GPP and POD0 in the beech and oak forests

**Figure 3:**
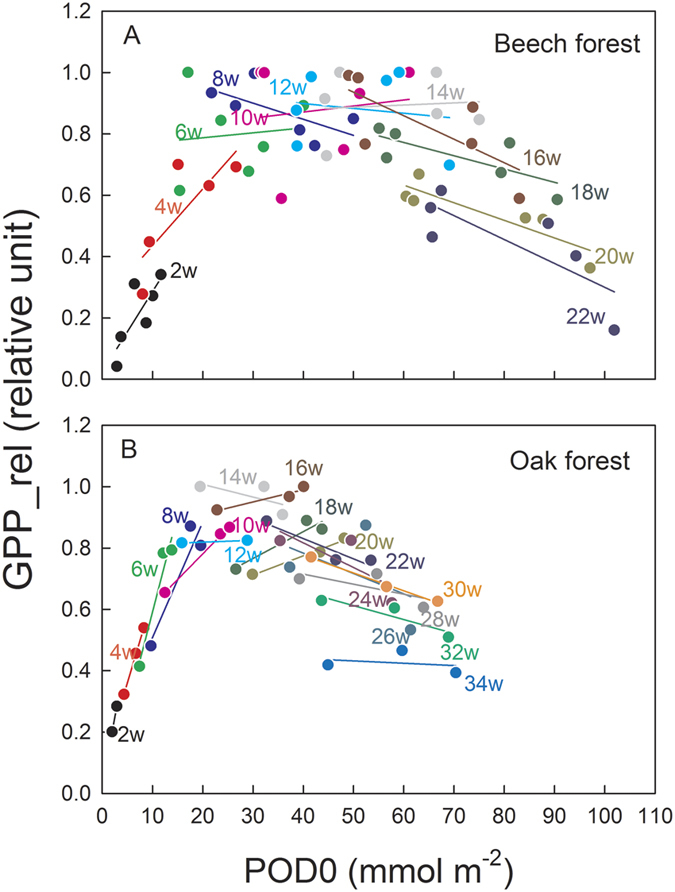
The relationship between the relative unit of light-saturated GPP (demonstrated as relative unit) and POD0 at the end of each period from the budbreak in the beech (**A**) and oak (**B**) forests. GPP_rel is calculated as follows: GPP_rel = (light-saturated GPP)/(maximum light-saturated GPP during the growth period for each year). The data are grouped by 2-week intervals from the budbreak demonstrated by different colors. Budbreaks in the beech forest occurred during April 30 to May 16, while those in the oak forest occurred during March 30 to April 8.

We investigated light-saturated GPP as a function of POD0 from the onset of budbreak. The maximum light-saturated GPP of the beech forest varied among the years; however, the environmental factors determining the inter-annual variation have not been fully identified^18^. A survey of each individual tree suggested that the oak forest was still growing as the total above-ground biomass [diameter at breast height (DBH) ≥3 cm] increased from 2004 to 2009: 108 and 126 Mg dw ha^−1^, respectively, estimated by allometric equations^22^. Therefore, to investigate the effects of O_3_ on the seasonal changes in foliar photosynthetic maturation and senescence, we used a relative unit of GPP (GPP_rel), which is calculated as follows: GPP_rel = (light-saturated GPP)/(maximum light-saturated GPP during the growth period for each year)^23^. The light-saturated GPP was estimated as the GPP at a photosynthetic photon flux density (PPFD) of 1500 μmol m^−2 ^s^−1^ on the basis of the GPP light-response curves derived from the pooled data of 2-week intervals from the budbreak. We categorized the growth seasons into spring–summer (April or May to July) for leaf maturation and summer–autumn (August to October or November) for leaf senescence stages. Based on the multiple regression analysis, GPP_rel in the spring–summer period could be explained by three factors: leaf age, photoperiod, and POD0 in the beech forest. In contrast, GPP_rel in the oak forest could be explained by photoperiod and POD0 (Fig. 3A,B, Table 1). GPP_rel in the summer–autumn period in the beech forest could be explained by three environmental factors: leaf age, leaf-to-air vapor pressure deficit (VPD), and POD0 (Fig. 3A, Table 1), where GPP_rel decreased with increasing leaf age, VPD, and POD0. In contrast, the decrease in GPP_rel in the summer–autumn period in the oak forest could be explained by a decrease in the air temperature and increases in precipitation and VPD (Fig. 3B, Table 1). Notably, the multiple regression model including precipitation could better explain the response of the summer–autumn GPP_rel in the oak forest as a whole; the coefficient of precipitation was not significantly different from 0 (p = 0.148, Table 1).

### The effect of POD0 on leaf longevity in the cool temperate beech forest

**Figure 4:**
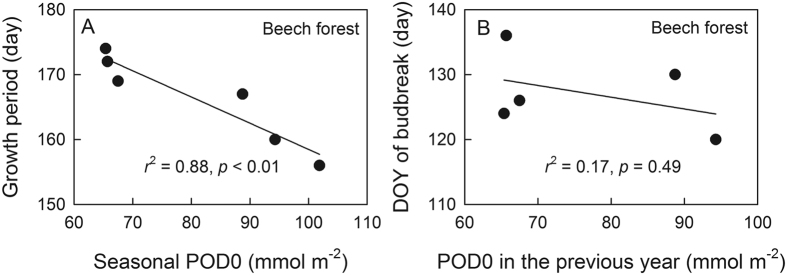
The relationship between the growth period and seasonal POD0 in the cool temperate beech forest (**A**). Growth period was defined when GPP > 0. Seasonal POD0 was set as POD0 for 22 weeks from the budbreak. The relationship between the day of year (DOY) of budbreak and seasonal POD0 in the previous year (**B**). DOYs of budbreak in 2002–2006 are plotted against POD0 in 2001–2005, respectively.

In our study, we defined leaf longevity as a growth period from budbreak until foliage senescence, when GPP > 0 in the beech forest^18^. However, the growth period for the oak trees was difficult to determine, because GPP did not become 0 due to the remaining evergreen species^19^. Leaf longevity was negatively correlated with higher POD0 in the beech forest during the growth season (Fig. 4A), although the date of leaf emergence was unaffected by POD0 in the previous year (Fig. 4B).

## Discussion

On the basis of the practical approach using photosynthesis-dependent stomatal model, we can estimate O_3_ fluxes continuously in the cool temperate deciduous forest and warm temperate mixed forest throughout the growth season even when the canopy was wet with rainfall or not fully closed during leaf expansion in spring and leaf shedding in autumn^17^. We found a time-lag between the peaks of O_3_ exposure and O_3_ flux, which suggests that higher O_3_ exposure would not necessarily result in the higher O_3_ flux. Thus, seasonal changes in G_s_ should be taken into account for a flux-based O_3_ assessment^13^,^14^. Konara oak has a relatively higher drought tolerance among the temperate forest tree species because it grows in dry habitats on mountain ridges with a conservative water use^21^,^24^. Consistently, the oak forest showed substantially lower G_s_ and POD0 than those of the beech forest, although the maximum leaf area index (LAI) was not significantly different (mean ± SD: 4.6 ± 0.2 and 4.9 ± 0.3, respectively).

Increased POD0 showed a positive effect on leaf maturation in both forests, besides photoperiod and leaf age, during the early growth season (Fig. 3A,B). As young leaves have generally less sensitivity to O_3_ as compared to old leaves^21^,^25^,^26^, and relatively higher photosynthetic rates were observed in immature leaves of Konara oak and Japanese oak (*Q. mongolica* var. *crispula*) grown under elevated O_3_^21^,^26^, a stimulating effect of O_3_ on photosynthesis at the early stage of leaf development warrants further investigation. In contrast, increased POD0, as well as VPD, may accelerate age-dependent leaf senescence in the O_3_-sensitive beech forest in the late growth season (Fig. 3A), whereas leaf senescence was not influenced by POD0 and was primarily influenced by air temperature and VPD in the O_3_-tolerant oak forest (Fig. 3B, Table 1). Lower O_3_ sensitivity during leaf senescence in the oak forest may be attributed to the lower POD0 due to the lower G_s_ (Fig. 2D,F), as reported for the seedlings of Konara oak grown under free-air O_3_ fumigation^21^. Furthermore, Konara oak is known as a typical isoprene-emitting species in Japan^27^,^28^, which probably scavenges O_3_ inside the leaf and contributes to detoxification^29^ or the enhancement of membrane functions against O_3_ injury^30^. Such a difference in intrinsic O_3_ tolerance might be involved in the canopy-level responses to O_3_ dose as well as the avoidance of O_3_ uptake via stomatal closure. The earlier autumn senescence demonstrated by the decline in GPP in the beech forest was also supported by the reduced growth period (defined as GPP > 0) (Fig. 4A)^2^,^31^,^32^,^33^,^34^. Although elevated O_3_ retards the onset of leaf emergence in birch trees (*Betula pendula*) in the next spring due to a decrease in the stored carbohydrates^35^, this was not the case in our study because no relationship was found between the date of leaf emergence and POD0 in the previous year (Fig. 4B). Because of snow cover on the forest floor for 5–6 months up to a height of 2 m in winter (November to April), and regular precipitation in late summer at the Appi forest site^18^, there might be little possibility that soil water deficit decreased the growth period. Furthermore, precipitation was excluded as an explanatory variable in the multiple linear regression, which partly supports that soil water content was not the factor regulating leaf senescence at the site.

Our findings demonstrate that the photosynthetic C sequestration capacity of the forest ecosystem is potentially affected even today in an O_3_-sensitive forest by the present-level O_3_ based on long-term CO_2_ flux observations. O_3_-induced earlier leaf senescence has been reported previously using trees grown in open-top chambers and free-air fumigation systems^2^,^32^,^33^,^34^. In our study, such an accelerated senescence by O_3_ was apparently detected in the O_3_-sensitive beech species at the real-world forest level. As the atmospheric O_3_ concentrations are predicted to increase, especially in East Asia, including Japan^1^,^2^,^3^,^4^, earlier leaf senescence and the consequent shorter growth period induced by elevated O_3_ would cause a further adverse effect on forest C sequestration in the future, especially in the forests consisting of O_3_-sensitive species.

## Methods

### Study sites

Appi forest meteorology research site (40°00′N, 140°56′E, 825 m a.s.l.) is located on the Appi highland in Iwate Prefecture, Japan (details are described in ref. 18). The site is located in a secondary cool temperate deciduous broadleaf forest, primarily consisting of the Japanese beech (*Fagus crenata* Blume), which is approximately 80 years old. The canopy height was measured to be 19–20 m in 2009. There is not much vegetation on the forest floor, and evergreen trees are rarely observed. It snows heavily from November to May, with a snow depth reaching 2 m. The annual mean temperature was 5.9 °C (in 2000–2006), annual precipitation was 1869 mm (in 2007–2009), and annual mean solar radiation was 12.7 MJ m^−2 ^day^−1^ (in 2000–2006). The soil has been classified as moderately moist brown forest soil.

Yamashiro forest hydrology research site (34°47′N, 135°50′E, 220 m a.s.l.) is situated in the southern part of Kyoto Prefecture, Japan (details are described in ref. 19). It is located in a warm temperate mixed deciduous and evergreen broadleaf forest, which is built upon weathered granite. After the invasion by pine wilt disease in 1980s, Konara oak (*Quercus serrata* Thunb. ex. Murray) has taken over and the forest is now regenerated. The tree biomass [diameter at breast height (DBH) ≥3 cm] was estimated at 96 Mg dw ha^−1^ in 1999, dominated by Konara oak classified as a deciduous broadleaf tree species (66% of biomass) and *Ilex pedunculosa* Miq. (an evergreen broadleaf tree species; 28% of biomass)^27^. The canopy height ranged from 6 m to 20 m with an average of 12 m. The annual mean temperature was 14.7 °C (in 2000–2002), annual precipitation was 1095 mm (in 2000–2002), and annual mean solar radiation was 11.9 MJ m^−2^ day^−1^ (in 2000–2002).

### Flux measurements over the forests

Meteorological towers were constructed in the forests to facilitate the measurement of fluxes of CO_2_, energy, and water vapor between the forest ecosystem and atmosphere^18^,^19^. The towers were equipped with a system for measuring eddy covariance (EC) that used a closed-path CO_2_ analyzer to measure the CO_2_ flux above the forest. The EC sensors were mounted on top of the tower. The EC was measured using a three-dimensional ultrasonic anemometer-thermometer (DAT-600-3TV, Kaijo, Tokyo, Japan) and a closed-path CO_2_ infrared gas analyzer (IRGA; LI-6262, Li-Cor, Lincoln, NE, USA). The sampling frequency was 10 Hz.

### Estimation of light-saturated gross primary production

The gross primary production (GPP) was estimated as follows: respiration of ecosystem (R_eco_) − net ecosystem exchange (NEE). Light-saturated GPP was derived from the relationship between GPP and photosynthetic photon flux density (PPFD). The data were rejected when the friction velocity (u*) was below 0.25 m s^−1^; however, in case of precipitation, the data were accepted. We regressed the relationship between GPP and PPFD as follows:


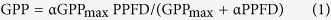


where α denotes the ecosystem quantum yield and GPP_max_ is the maximum GPP. We derived α and GPP_max_ for the pooled data at 2-week intervals from the onset of budbreak. We set the GPP at a PPFD of 1500 μmol m^−2 ^s^−1^ based on the equation as the light-saturated GPP (Tables S1 and S2).

The maximum light-saturated GPP of the beech forest varied among the years; however, the environmental factors determining the inter-annual variation have not been fully identified^16^. A survey of each individual tree suggested that the oak forest was still growing as the total biomass increased from 2004 to 2009. Therefore, to investigate the effects of O_3_ on the seasonal changes in foliar photosynthetic maturation and senescence, we used a relative unit of GPP (GPP_rel), which is calculated as follows: GPP_rel = (light-saturated GPP)/(maximum light-saturated GPP during the growth period for each year)^23^. The light-saturated GPP was estimated as the GPP at a PPFD of 1500 μmol m^−2 ^s^−1^ based on the GPP light-response curves derived from the pooled data of 2-week intervals from the budbreak. We categorized the growth seasons into spring–summer (April or May to July) for leaf maturation and summer–autumn (August to October or November) for leaf senescence stages.

### Estimation of phytotoxic O_3_ dose above a threshold of 0 uptake (POD0)

The estimation of stomatal O_3_ fluxes involves several steps^36^,^37^. The aerodynamic resistance (R_a_) is calculated from measured micrometeorological parameters such as friction velocity and sensible heat flux by using the Monin–Obukhov theory (see ref. 37, for calculation details), whereas the quasi-laminar layer resistance (R_b_) is calculated by using the parameterization proposed previously^38^. We calculated the surface resistance (R_c_) from the stomatal (R_ST_) and non-stomatal resistance for O_3_ (R_NS_) as follows:


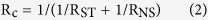



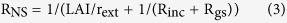


where LAI is the leaf area index (m^2 ^m^−2^), r_ext_ denotes the external leaf resistance; R_inc_, the in-canopy resistance; and R_gs_, the ground surface resistance. The maximum LAI in the beech forest was estimated from the amounts of leaf litter^18^. We assumed that the LAI of the beech forest increased linearly from 0 to the maximum within 1 month from the budbreak, and then decreased from the maximum to 0 also within 1 month before the end of the foliage period based on the field observations. In contrast, seasonal changes in the LAI in the oak forest were measured using a plant canopy analyzer (LAI-2000; Li-Cor, Lincoln, NE, USA). The external leaf resistance (r_ext_) is set at 2500 s m^−1^, the in-canopy resistance (R_inc_) is defined as b LAI h/u*, where h is the canopy height and b is an empirical constant taken as 14 m^−1^, and R_gs_ is set at 200 s m^−1^ 39,40.

The stomatal O_3_ flux (F_ST_) is obtained as follows:


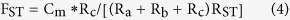


where C_m_ denotes the ozone concentration at the measurement height. The stomatal resistance (R_ST_) is calculated by multiplying the stomatal resistance for water vapor flux (noted as R_s_) by 1.65^37^. R_s_ is calculated from the water vapor flux (λE) by using the Penman–Monteith equation^41^. The measured canopy-level stomatal conductance (G_s_) is expressed as 1/R_s_. O_3_ data were monitored at the top of the flux towers during the growth seasons in 2012 and 2013. We compared them with the O_3_ observations at the adjacent air pollution stations; Tsushida (Morioka city), 43 km SSE of Appi forest site, and Tanabe (Kyotanabe city), 8 km ENE of Yamashiro forest site, respectively. Then, we developed a multiple linear regression model for each forest site estimating the daytime tower O_3_ concentration, using O_3_ and NO_2_ concentrations at the air pollution station and air temperatures at both the station and the forest site (details in ref. 42). Using the models, we estimated C_m_ at the flux sites (2001 to 2006 at Appi forest site and 2004, 2005, and 2009 at Yamashiro forest site) based on the past meteorological data monitored at the adjacent air pollution stations.

As the G_s_ estimation from the Penman–Monteith equation is valid only for a dry and closed canopy^15^, to estimate G_s_ continuously, we used the modified Ball–Woodrow–Berry model^43^, taking the nonlinear response of G_s_ to relative humidity into account^17^,^44^. We estimated G_s_ as follows:


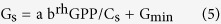


where G_min_ denotes the minimum conductance in the dark; a and b, the empirical scaling parameters; GPP, the gross primary production; rh, the relative humidity; and C_s_, the leaf surface CO_2_ concentration. We determined the coefficients in the equation using G_s_ derived by the Penman–Monteith equation when the canopy was closed from June to August in the beech forest, and from June to September in the oak forest without rain (>1 mm within 24 h) for each year (details are shown in ref. 17). An uncoupling between photosynthesis and stomatal conductance induced by O_3_ exposure has been reported in the previous studies^32^,^45^,^46^. Decrease in the stomatal conductance at a given photosynthetic rate was observed in adult beech trees (*Fagus sylvatica*) exposed to elevated O_3_ during early and late growth seasons (at the end of June and August, respectively)^32^,^45^. In the present study, although seasonally-investigated O_3_ effects on stomatal conductance were not reflected in the G_s_ estimation, yearly differences in the O_3_-induced uncoupling were taken into account. In contrast, stomatal sluggishness, i.e., losing precise regulation of stomata, sometimes observed at the end of the growth season^46^, was not taken into account in the present study. In the case of leaf sluggishness at the end of the growth season, it should be noted that the estimation of G_s_ using the BWB model described above might be underestimated.

The POD0 in the forest ecosystem was calculated by summing up the ozone stomatal flux (F_ST_) during the daytime (PPFD > 0) from the budbreak to a given period as follows:


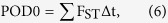


where the mean daily sum of F_ST_ for the rest days of each month was substituted for days when F_ST_ data were unavailable.

### Statistical analysis

Multiple regression analysis was used for a quantitative evaluation of the influence of the major explanatory factor(s) on the photosynthetic capacity. We initially set six explanatory factors affecting potential photosynthetic performance (GPP_rel): leaf age, photoperiod, air temperature, precipitation, leaf-to-air vapor pressure deficit (VPD) and POD0^20^. VPD was defined as the difference between the saturated vapor pressure at the canopy surface temperature and the vapor pressure of the ambient air. The surface temperature (t_s_) was estimated as follows:


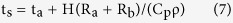


where t_a_ is the air temperature at the top of tower, H is the sensible heat flux estimated by EC, C_p_ is the heat capacity of air, ρ is the density of air^15^. All the variables were standardized to a mean of 0 and a variance of 1 prior to modeling to quantitatively evaluate the influence of the major explanatory factors. Stepwise regressions were undertaken to define the subset of effects that would altogether provide the smallest corrected Akaike information criterion (AICc) in subsequent modeling. Furthermore, we also calculated variance inflation factor (VIF) as a measure of multicollinearity. We considered that VIF greater than 5 constitutes a multicollinearity problem. In case of VIF(s) > 5, we removed the variable with the single highest VIF and then recalculated stepwise regressions using the remaining variables until all VIFs fell below 5.

## Additional Information

**How to cite this article**: Kitao, M. *et al.* Increased phytotoxic O_3_ dose accelerates autumn senescence in an O_3_-sensitive beech forest even under the present-level O_3_. *Sci. Rep.* **6**, 32549; doi: 10.1038/srep32549 (2016).

## Supplementary Material

Supplementary Information

## Figures and Tables

**Figure 1:**
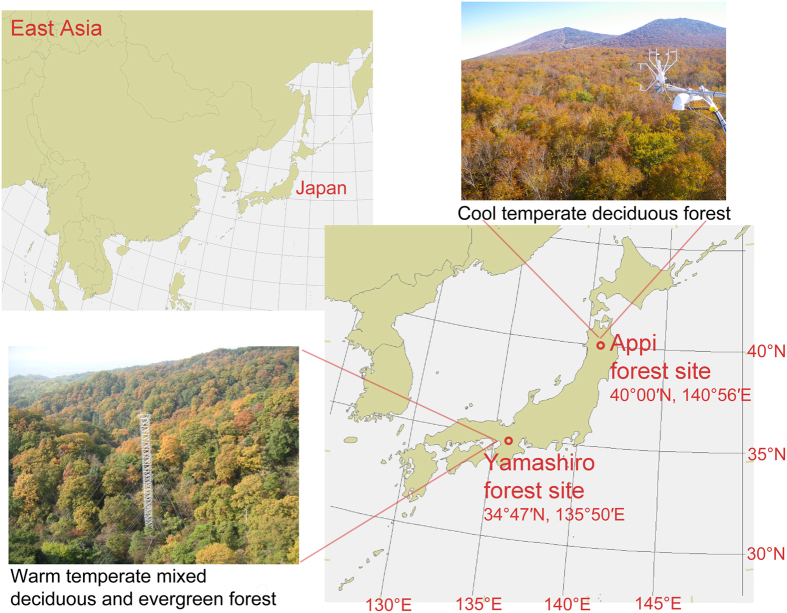
Locations of the cool temperate forest consisting of beech trees at Appi forest site and the warm temperate mixed deciduous and evergreen forest dominated by oak trees at Yamashiro forest site. Maps are created by Dr. I. Tsuyama using ESRI ArcGIS (v9.3, http://www.esri.com/software/arcgis/arcgis-for-desktop).

**Table 1:** Summary of multiple linear regressions relating photosynthetic activity to environmental factors.

Forest type	Dependent variable	Summary measures		Regression coefficients			
		*R*^*2*^	*P*	Independent variable	Coefficients	*P*	VIF
Beech forest	GPP_rel (leaf maturation; spring–summer)	0.80 (n = 35)	<0.001	Leaf age	0.446	0.003	3.30
				Photoperiod	0.415	<0.001	1.01
				POD0	0.359	0.014	3.29
	GPP_rel (leaf senescence; summer–autumn)	0.76 (n = 33)	<0.001	Leaf age	−0.687	<0.001	1.21
				VPD	−0.230	0.012	1.21
				POD0	−0.453	<0.001	1.37
Oak forest	GPP_rel (leaf maturation: spring–summer)	0.80 (n = 22)	<0.001	Photoperiod	0.371	0.0102	1.75
				POD0	0.616	<0.001	1.75
	GPP_rel (leaf senescence; summer–autumn)	0.63 (n = 27)	<0.001	Air temperature	1.314	<0.001	4.16
				Precipitation	−0.193	0.148	1.41
				VPD	−0.613	0.015	4.55

Initial explanatory factors affecting photosynthetic performance: leaf age, photoperiod, air temperature, precipitation, VPD and POD0. All the variables were standardized to a mean of 0 and a variance of 1 prior to modeling to quantitatively evaluate the influence of the major explanatory factors. Stepwise regressions were undertaken to define the subset of effects that would altogether provide the smallest corrected Akaike information criterion (AICc) in subsequent modeling. As a measure of multicollinearity, variance inflation factor (VIF) is demonstrated. We considered that VIF greater than 5 constitutes a multicollinearity problem. In case of VIF(s) > 5, we removed the variable with the single highest VIF and then recalculated stepwise regressions using the remaining variables until all VIFs fell below 5.
