# Supplementary material for: Increased phytotoxic O3 dose accelerates autumn senescence in an O3-sensitive beech forest even under the present-level O3
Source: Sci Rep. 2016 Sep 7;6:32549. doi: 10.1038/srep32549 (PMC5013268; doi:10.1038/srep32549)
Supplement: Supplementary Information [file srep32549-s1.pdf]

Increased phytotoxic O_3 dose accelerates autumn senescence in an O_3-sensitive beech forest even under the present-level O_3

Mitsutoshi Kitao, Yukio Yasuda, Yuji Kominami, Katsumi Yamanoi, Masabumi Komatsu, Takafumi Miyama, Yasuko Mizoguchi, Satoshi Kitaoka, Kenichi Yazaki, Hiroyuki Tobita, Kenichi Yoshimura, Takayoshi Koike & Takeshi Izuta

Supplementary Table S1. Light-saturated GPP in the beech forest for each period at 2-week intervals from the budbreak

Weeks from the budbreak	Light-saturated GPP (μmol m^{-2} s^{-1})					
	2001	2002	2003	2004	2005	2006
0–2	9.0	4.2	6.8	8.4	1.4	11.6
3–4	21.0	13.7	23.2	18.9	9.0	23.6
5–6	22.5	18.9	27.9	22.7	32.4	30.4
7–8	27.0	28.6	28.0	26.8	28.9	28.9
9–10	24.9	30.7	34.3	15.9	32.4	34.0
11–12	32.4	26.9	36.8	26.6	24.6	23.8
13–14	33.3	28.0	31.9	26.9	23.6	28.8
15–16	29.5	30.4	28.3	20.7	31.8	20.1
17–18	25.6	25.1	24.8	21.6	23.4	19.9
19–20	17.3	18.3	19.3	18.0	18.9	12.3
21–22	13.4	17.2	18.7	16.6	15.0	5.5

Supplementary Table S2. Light-saturated GPP in the oak forest for each period at 2-week intervals from the budbreak. NA indicates that data were unavailable. The datasets from 2006 to 2008 are unavailable at present.

Weeks from the budbreak	Light-saturated GPP (μmol m^{-2} s^{-1})		
	2004	2005	2009
0–2	4.3	5.6	NA
3–4	6.9	9.0	12.0
5–6	8.9	15.4	17.7
7–8	10.3	17.1	18.0
9–10	14.1	16.6	19.3
11–12	17.6	16.2	NA
13–14	21.5	19.7	20.3
15–16	19.9	19.0	22.3
17–18	15.7	17.5	19.2
19–20	15.3	15.5	18.5
21–22	19.1	15.0	16.9
23–24	17.7	16.2	13.8
25–26	15.9	17.2	11.9
27–28	15.0	14.1	13.5
29–30	16.6	13.2	14.0
31–32	13.5	11.9	11.3
33–34	9.0	9.1	8.8
